# Mapping the correlations and gaps in studies of complex life histories

**DOI:** 10.1002/ece3.9809

**Published:** 2023-02-17

**Authors:** Emily L. Richardson, Dustin J. Marshall

**Affiliations:** ^1^ Centre for Geometric Biology, School of Biological Sciences Monash University Melbourne Victoria Australia

**Keywords:** carry‐over effects, complex life histories, literature map, longitudinal studies, Simpson's paradox

## Abstract

For species with complex life histories, phenotypic correlations between life‐history stages constrain both ecological and evolutionary trajectories. Studies that seek to understand correlations across the life history differ greatly in their experimental approach: some follow individuals (“individual longitudinal”), while others follow cohorts (“cohort longitudinal”). Cohort longitudinal studies risk confounding results through Simpson's Paradox, where correlations observed at the cohort level do not match that of the individual level. Individual longitudinal studies are laborious in comparison, but provide a more reliable test of correlations across life‐history stages. Our understanding of the prevalence, strength, and direction of phenotypic correlations depends on the approaches that we use, but the relative representation of different approaches remains unknown. Using marine invertebrates as a model group, we used a formal, systematic literature map to screen 17,000+ papers studying complex life histories, and characterized the study type (i.e., cohort longitudinal, individual longitudinal, or single stage), as well as other factors. For 3315 experiments from 1716 articles, 67% focused on a single stage, 31% were cohort longitudinal and just 1.7% used an individual longitudinal approach. While life‐history stages have been studied extensively, we suggest that the field prioritize individual longitudinal studies to understand the phenotypic correlations among stages.

## Graphical abstract

When testing for phenotypic correlations between stages in complex life cycles, using an experimental design that follows cohorts of individuals through multiple stages is common however, this design is at risk of Simpson's Paradox, where patterns observed at the cohort level do not reflect patterns observed at the individual level. We conducted a systematic literature map on marine invertebrate life histories, and of +3000 experiments, just 1.7% followed individuals through multiple stages, a method that removes the risk of Simpson's Paradox. Our results suggest our knowledge of phenotypic correlations between stages is limited, highlighting the enormous scope for future studies on complex life histories.

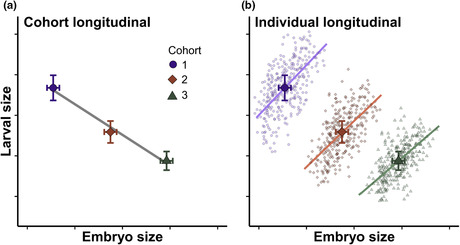

## 1 INTRODUCTION

Complex life cycles, whereby organisms pass through distinct life‐history stages before reaching the adult stage, are ubiquitous in the animal kingdom. While life‐history stages can be quite different from each other in terms of morphology, trophic mode and even habitat, phenotypic and genetic correlations between life‐history stages are ecologically and evolutionarily important. For example, larval size and size at metamorphosis are positively correlated in frogs (Relyea, 2001), marine invertebrates (Trackenberg et al., 2020), and insects (Tammaru, 1998; Tammaru et al., 2004). If traits are correlated across stages, an early‐life experience may determine performance later in the life history (i.e., “carry‐over” or “latent’ effects Pechenik, 2006) for example, juvenile body size and/or growth can be affected by temperature (moths, Galarza et al., 2019; sticklebacks, Kim et al., 2018), food availability (mussels, Phillips, 2002; frogs, Warne & Crespi, 2015), and season length (frogs, Prokic et al., 2021; frogs, Szekely et al., 2020; damselflies, Tuzun & Stoks, 2018;) of the larval stage. Such phenotypic correlations can have cascading effects on population dynamics (Burgess & Marshall, 2011; Taylor & Scott, 1997).

Phenotypic correlations among life‐history stages also affect evolutionary trajectories. The degree to which phenotypes in different life‐history stages are evolutionarily independent remains the subject of intense discussion (e.g., the “adaptive decoupling hypothesis,” Bonett & Blair, 2017; Ebenman, 1992; Moran, 1994; Sherratt et al., 2017). When traits among life‐history stages are correlated, each stage can constrain the other from evolving to its optimum (Aguirre et al., 2014; Marshall & Morgan, 2011). The broad interest in understanding correlations across life‐history stages has generated a wealth of empirical work (Harrison et al., 2011; Moore & Martin, 2021; O'Connor et al., 2014; Pechenik et al., 1998; Pechenik, 2006) but our understanding of correlations among certain life‐history stages remains incomplete. An important step in developing the field and identifying priorities is to identify the aspects of complex life histories that have been relatively well studied, and to locate any knowledge gaps that remain.

Currently, it remains unclear whether all life‐history stages have been studied equally or if emphases on particular stages exist. For example, an informal reading of literature implies that correlations between the larval and juvenile stages are of particular interest in studies of marine invertebrates (Mendt & Gosselin, 2021; Phillips, 2002), insects (Carter & Sheldon, 2020; Moore, 2021; Moore and Martin, 2021), frogs (Green & Bailey, 2015; van Allen et al., 2010), and fish (Araki et al., 2009; Dingeldein & White, 2016). However, by focusing on a few stages, we may be missing key correlations that could regulate populations—for example, the egg stage may determine adult phenotypes and densities, and the effect of this correlation can last for several generations (Downes et al., 2021; Plaistow et al., 2006). Identifying emphases in the literature would allow future studies to address which stages/correlations are less examined, and provide a more complete understanding of complex life histories and their evolutionary trajectories, such that no one stage remains a “black box.”

**FIGURE 1:**
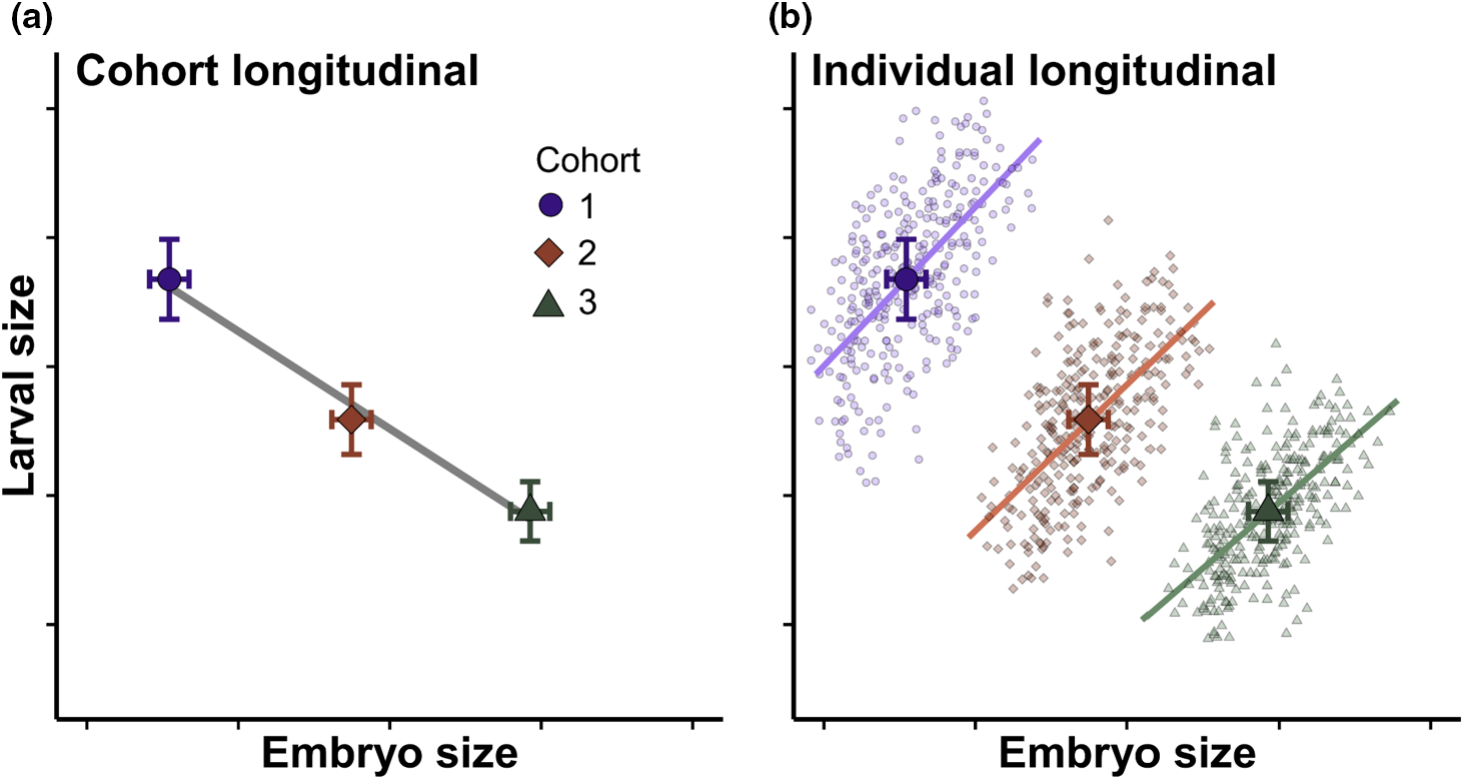
Hypothetical data depicting experimental design approaches used to study life histories (single stage studies not shown). (a) Cohort longitudinal studies follow groups (i.e., “cohorts”) of individuals across stages. Points represent means ± SE for larval and adult traits of three cohorts. (b) Individual longitudinal studies follow individuals through multiple stages. A small point is an individual, and large points are the means ± SE of each cohort, depicted in panel a. The panels show an example of Simpson's Paradox, where the relationship between two traits across cohorts (a) is the opposite of the relationship observed across individuals within each cohort (b). To make inferences about phenotypic correlations among life‐history stages within a species, the individual longitudinal approach is most appropriate.

We also must consider how we study the life cycle—different experimental designs for studying life histories provide access to different inferences. Broadly, there are three experimental design approaches: (1) single stage, (2) cohort longitudinal, and (3) individual longitudinal. Single stage studies are useful for describing traits in one particular stage of the life history, or for estimating covariances across species, but because they only examine one stage, they tell us nothing about phenotypic correlations within species. Longitudinal approaches allow us to make inferences about correlations—traits are measured in multiple stages, so (co)variances between stages can be estimated. Even then, the two longitudinal approaches have different strengths and weaknesses. In cohort longitudinal approaches, groups of individuals or “cohorts” are reared through the life history (Figure 1a). Cohort longitudinal studies are often less finicky than individual longitudinal studies, particularly for very small organisms or those with high mortality rates. If one is interested in quantifying *genetic* correlations among life‐history stages, and a quantitative genetics breeding design is used, the scale of replication is cohort from a single sire, and therefore cohort longitudinal approaches are appropriate (e.g., Aguirre et al., 2014). But cohort longitudinal studies are susceptible to Simpson's Paradox whereby trait relationships observed across cohorts may not reflect the trait relationships for individuals; the trend at the individual level could even be opposite of that at the cohort level (Figure 1; note that among‐species comparisons are also vulnerable to Simpson's Paradox). Thus, if one wishes to make inferences about *phenotypic* correlations among life‐history stages, then individual longitudinal studies (i.e., following individuals) are most suitable, as they estimate trait (co)variances at the appropriate scale and avoid the potential for Simpson's Paradox (Figure 1b). However, individual longitudinal studies are potentially laborious—rearing individuals can be much harder than rearing a cohort, so we might expect individual longitudinal studies to be rare. Identifying the relative prevalence of each experimental design approach will help to identify the state of our knowledge and our capacity to make conclusions about correlations across the life history and the degree to which we are at risk of Simpson's Paradox (i.e., cohort longitudinal studies).

Another component of experimental design that must be considered is where the study is conducted. The nature, strength, and variability of correlations between life‐history stages vary systematically between laboratory‐based study and those done in the field (Monro et al., 2010). Controlled laboratory conditions make experimental manipulations easier and are sometimes the only way to examine certain life‐history stages (Diamond, 1983). Nevertheless, field experiments provide information that cannot be gained from laboratory experiments alone (Reznick & Ghalambor, 2005). Estimating the ubiquity of field and laboratory studies should allow us to identify when field studies should be priority.

**FIGURE 2:**
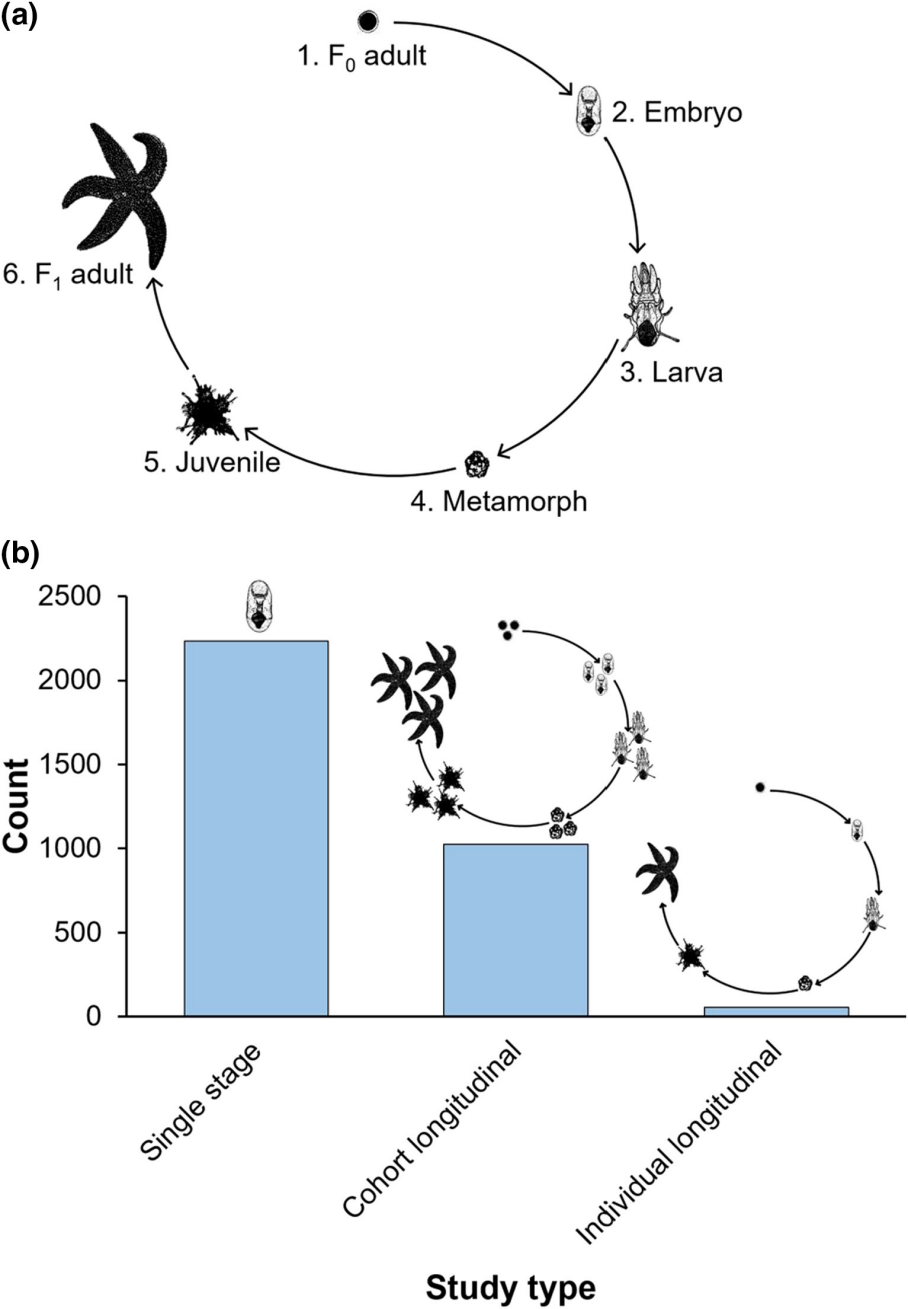
(a) Stages included in literature map of marine invertebrate life histories. For each study, the life‐history stages measured were recorded: (1) F_0_ adult; (2) embryo; (3) larva; (4) metamorph; (5) juvenile; and (6) F_1_ adult. (b) Frequency of each experimental design type identified in the literature map (*n* = 3315 studies total). Study type is depicted in the life cycle above each column—single stage studies measure one stage in the life history; cohort longitudinal studies follow groups (i.e., “cohorts”) of individuals across stages; and individual longitudinal studies follow individuals through multiple stages.

Here, we use a systematic map of the empirical literature on marine invertebrates to describe the state of our knowledge regarding phenotypic correlations across life‐history stages, and outline the field's strengths and knowledge gaps. Systematic maps use a repeatable methodological framework to quantify what has been studied. Unlike systematic reviews and meta‐analyses, systematic maps do not statistically analyze combined data from empirical studies (James et al., 2016; O'Dea et al., 2021). Instead, a systematic map collates, catalogues, and describes studies, outlining the current state of knowledge for a particular topic (James et al., 2016). Marine invertebrates are a good model group for mapping the current knowledge around correlations because of their numerous phyla, diverse life‐history modes, and long history of study from the perspective of complex life cycles (MacBride, 1907; Mortensen, 1923; Thorson, 1950). We collected methodological data for studies of life histories across the following six stages: (1) F_0_ adult, (2) embryo, (3) larva, (4) metamorph, (5) juvenile, and (6) F_1_ adult (Figure 2a) and recorded the experimental design used for each study.

### 1.1 Objectives

The objective of this systematic map was to determine areas of focus in life‐history studies using marine invertebrates as a model system.

The primary questions were as follows:

- What life‐history stages are most commonly studied?
- What experimental design approaches (i.e., single stage, cohort longitudinal, and individual longitudinal) are used?
- Are studies conducted in the laboratory or the field?
- Are there taxonomic emphases?

## 2 METHODS

### 2.1 Review team, inclusion criteria, and search strategy

We followed the six‐stage Systematic Mapping Methodology (James et al., 2016). The review team consisted of four primary reviewers and one stakeholder—the stakeholder commissioned and shaped the scope of the systematic map; established search methods and inclusion criteria; and contributed to data visualization. The four reviewers—one in 2014, two in 2015, and one in 2021, searched for and screened articles, and extracted and synthesized data.

We first established inclusion and exclusion criteria—life‐history studies had to be empirical, use marine invertebrates, and measure at least one fitness‐related trait (e.g., fecundity, survival, size, growth, development time)—for studies using more than one life‐history stage, the traits measured in each stage need not be the same (Tables A1 and A2). We excluded theoretical, observational, and qualitative studies without empirical data, and empirical studies that did not measure a fitness trait (e.g., behavior, metabolism, etc.).

To establish a search protocol, we first scoped Web of Science using the simple search function to identify search terms that would yield ~10% of articles to be accepted for the map (for search terms that were tested but not used see Table A3). Our protocol was to use chosen search terms to screen articles at the title and abstract level, and then import those that were relevant to EndNote (version X8.2). We then assessed the relevant full‐text articles for eligibility—once articles were approved for the map, data were extracted and added to a database in Excel 2016.

### 2.2 Searching, identification, and screening

Because we conducted four searches over 7 years, we had to slightly adjust our protocol each time to ensure that we were getting an unbiased sample of the literature, while still using methods that were logistically attainable (i.e., search terms that yielded a reasonable number of articles to be assessed). We used the four search strings provided in Table A4—all databases in Web of Science were used for searches 1–3, but only the Web of Science Core Collection was used for search 4 because searching all databases yielded too many articles for screening (>34,000 hits). In searches 1 and 2, we restricted the search to the journals *Biological Bulletin* and *Marine Biology*, respectively—we wanted to sample these journals because they have historically published studies on marine invertebrate life histories. For the other searches, all journals in the Web of Science were included. All hits were screened at the title and abstract level, except or the search 2—we only assessed the first 1000 articles, because relevant articles were scarce thereafter.

### 2.3 Coding and production of the map database

For the 1716 articles included, we recorded information on (1) species; (2) studies; and (3) references, and describe them below. Some articles had multiple studies, and thus have multiple rows in the database. Records were coded as having multiple studies if (1) more than one stage was investigated, but the stages were measured at different times and/or in different locations (e.g., two single stage studies for larvae and juveniles), or (2) if the same stage(s) were investigated in separate studies (e.g., one larval study that manipulates temperature and the other salinity). In total, we had data for 3315 studies.

#### 2.3.1 Species

We included phylum, class, and species for each study. We also recorded developmental mode: planktotrophic (i.e., planktonic, feeding larvae), lecithotrophic (i.e., planktonic, non‐feeding larvae), or direct development (i.e., aplanktonic, crawl‐away juveniles). We used the package “taxize” (Chamberlain and Szocs 2013) in R (v. 4.1.2) to search the Global Biodiversity Information Facility (GBIF) database to identify species names in the dataset that were synonyms—we refer to each species using just one name.

#### 2.3.2 Studies

For each study, we recorded the fitness traits measured for the life‐history stages: F_0_ adult, embryo, larva, metamorph, juvenile, and F_1_ adult (Table A2; Figure 2a). We classified each study into one of three experimental designs: single stage, cohort longitudinal (i.e., multiple stages following cohorts), and individual longitudinal (i.e., multiple stages following individuals). We also recorded whether the study was conducted in the field or laboratory. Studies conducted partially in the laboratory and field were classified overall as field studies because quantifying phenotypes almost inevitably required some laboratory work.

#### References

2.3.3

The full citation for each study is included.

After all data were extracted, reviewer 4 screened the entire map database to check for consistency and clarity across reviewers, meaning searches from 2014 and 2015 were double‐screened and are consistent with the 2021 search.

### 2.4 Data analysis and visualization

Coded data were analyzed in Excel 2016. Histograms were made in Excel 2016. Figures showing what stages cohort and individual longitudinal studies measured were made in PowerPoint 2016 (e.g., Figure 4).

## 3 RESULTS

**FIGURE 3:**
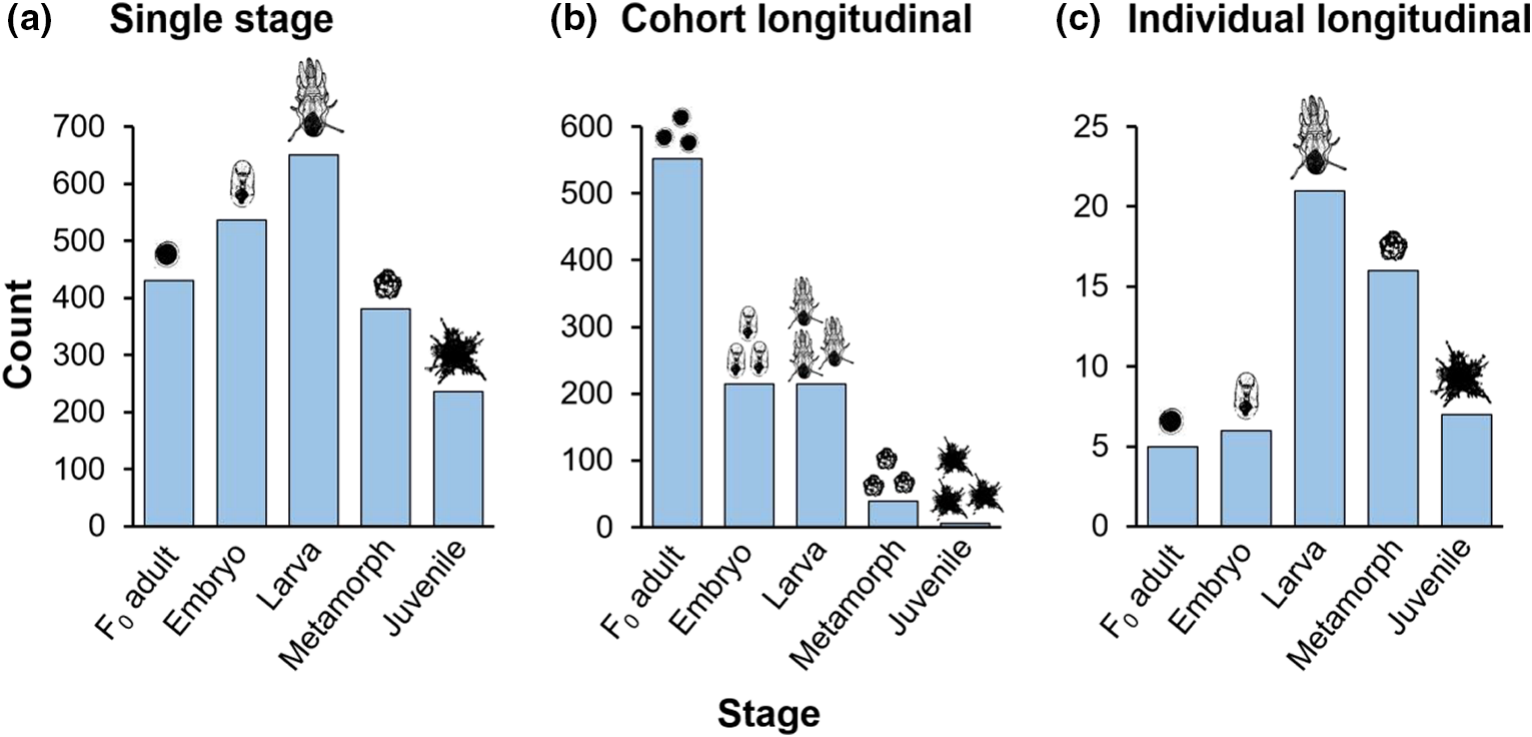
Frequency of studies that begin with each life‐history stage, across the three experimental design approaches: (a) single stage; (b) cohort longitudinal (i.e., multiple stages following groups of individuals); (c) individual longitudinal (i.e., multiple stages following individuals). Note the different scales on the *y*‐axes.

Of the 3315 studies in the dataset, 30.9% followed cohorts and only 1.7% followed individuals through multiple stages of the life cycle (Figure 2b). Studies were most commonly conducted in the laboratory (88.2%) and focused on a single stage (67.4%; Figure 2b). Studies beginning with the metamorph or juvenile stages were rare across all experimental design methods—generally, studies most often began with measuring F_0_ adults, embryos, and larvae (Figure 3).

**FIGURE 4:**
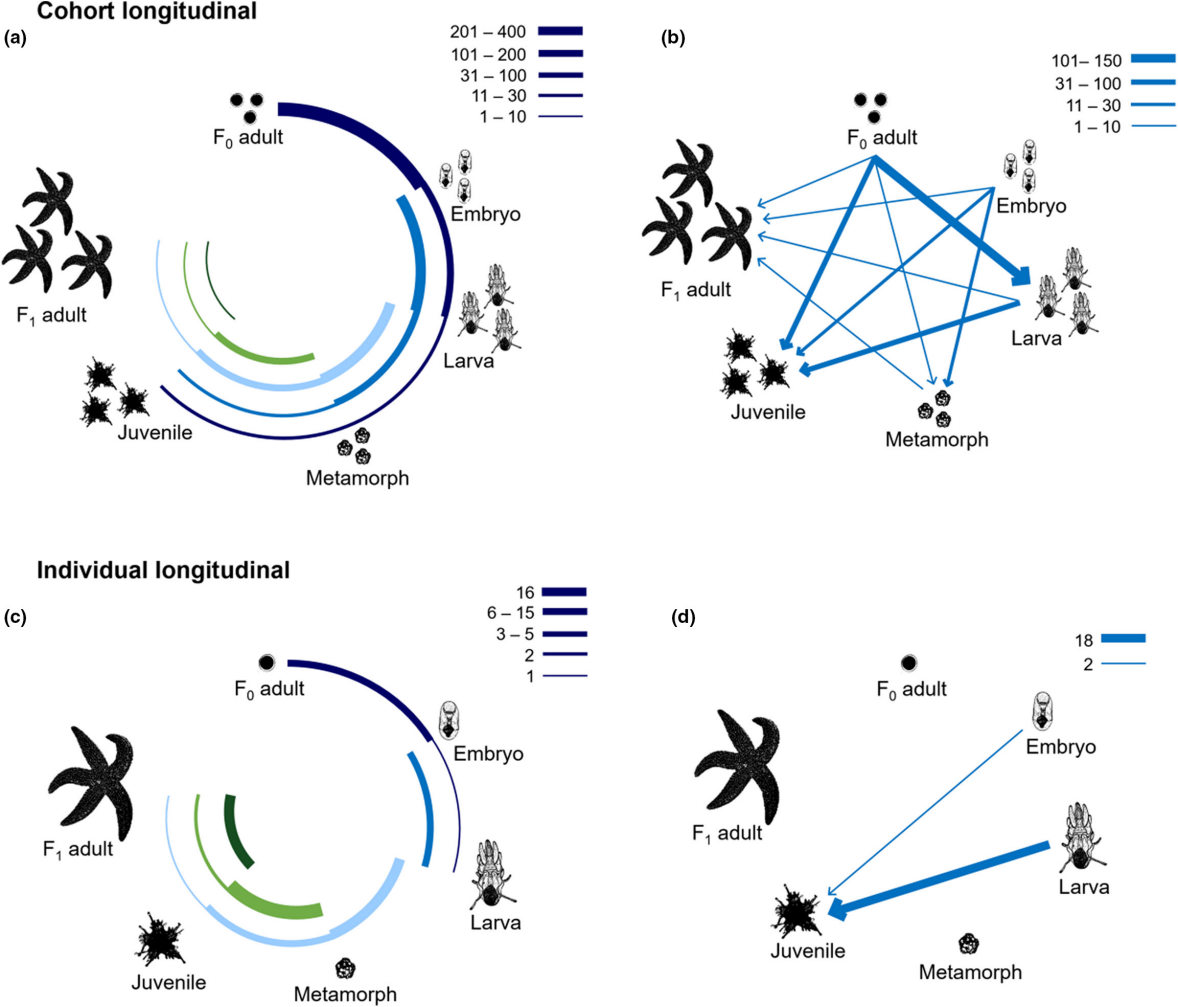
Summary of life‐history stages measured in (a, b) cohort longitudinal studies (i.e., multiple stages measured, following groups of individuals) and (c, d) individual longitudinal studies (i.e., multiple stages measured, following individuals). Thickness of bars represents the number of studies that measured each stage combination (see legends). (a, c) Studies that measure multiple stages sequentially. Concentric circles show studies that start at each stage—bars are thick at the start of each circle, and become narrow as fewer studies measure later stages. (b, d) Studies that measure multiple stages, but not sequentially. Lines show when studies skip stages (e.g., d; thick line represents studies that measured larvae and juveniles, but not metamorphs).

When we quantified what stages the longitudinal approaches measured the most, we found that studies following cohorts mostly measured the F_0_ adult, embryo, and larval stages (Figure 4a,b), whereas individual longitudinal studies focused on juveniles and F_1_ adult stages (Figure 4c,d). Most studies measured stages sequentially (i.e., did not skip stages, Figure 4b,d), but across all experimental design approaches, no study measured a trait in all six stages of the life cycle.

**FIGURE A1:**
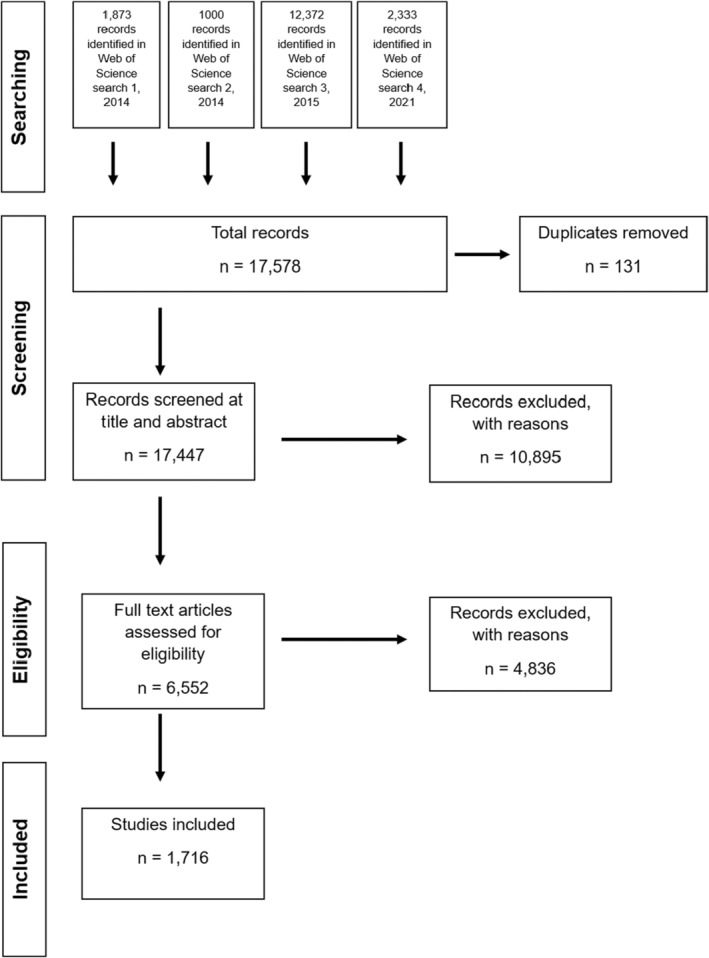
Flow diagram showing the selection process for articles included in the systematic map of marine invertebrate life histories. The searching, screening, eligibility, and inclusion processes were adapted from the Systematic Mapping Methodology (James et al., 2016). At the top, the number of articles identified from each of the four searches is provided—details of the four searches, including search terms used, are provided in Table A4. After 131 duplicates were removed, 17,447 were screened at the title and abstract level, 6552 full‐text articles were checked for eligibility, and ultimately 1716 articles were approved for the final literature map.

**FIGURE A2:**
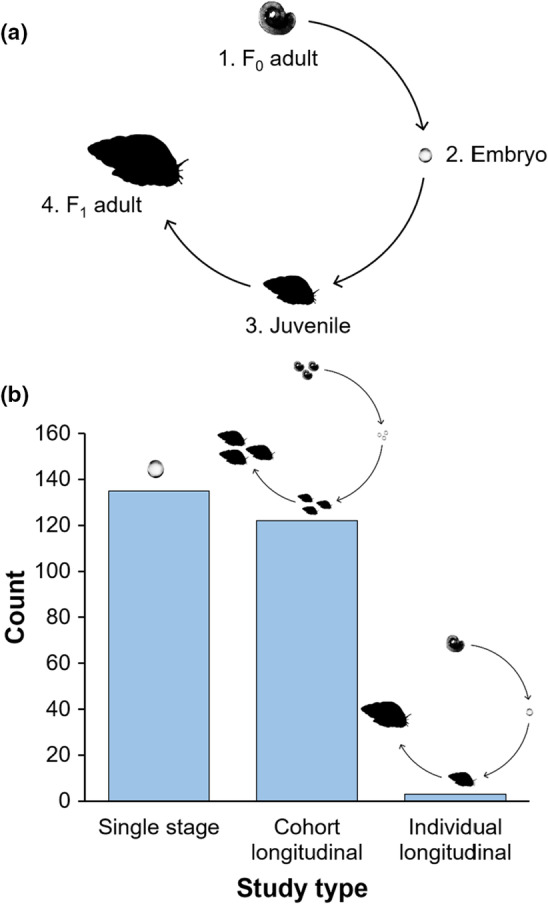
(a) Stages included in literature map of marine invertebrate life histories for species with direct development. For each study, the life‐history stages measured were recorded: (1) F_0_ adult; (2) embryo; (3) juvenile; and (4) F_1_ adult. (b) Frequency of each experimental design type identified in the literature map (*n* = 260 studies total). Study type is depicted in the life cycle above each column—single stage studies measure one stage in the life history; cohort longitudinal studies follow groups (i.e., “cohorts”) of individuals across stages; and individual longitudinal studies follow individuals through multiple stages.

**FIGURE A3:**
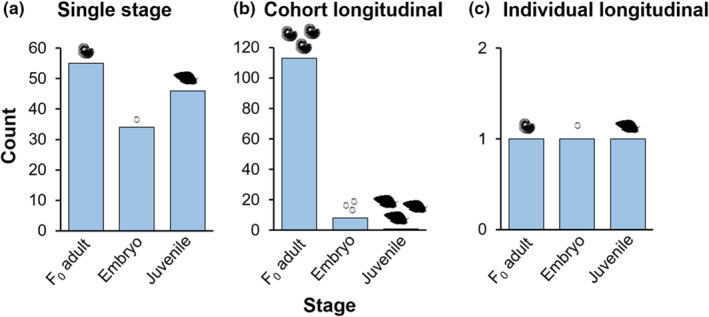
Frequency of studies that begin with each life‐history stage, across the three experimental design approaches: (a) single stage; (b) cohort longitudinal (i.e., multiple stages following groups of individuals); (c) individual longitudinal (i.e., multiple stages following individuals).

**FIGURE A4:**
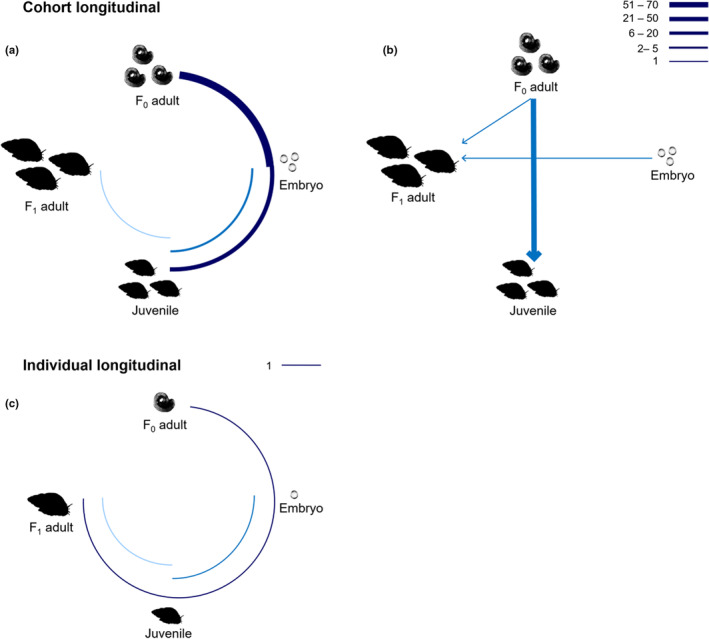
Summary of life‐history stages measured in (a, b) cohort longitudinal studies (i.e., multiple stages measured, following groups of individuals) and (c, d) individual longitudinal studies (i.e., multiple stages measured, following individuals). Thickness of bars represents the number of studies that measured each stage combination (see legends). (a, c) Concentric circles show studies that start at each stage—bars are thick at the start of each circle, and become narrow as fewer studies measure subsequent stages. (b) Lines show when studies skip stages (e.g., thick line represents studies that measured F_0_ adults and juveniles, but not embryos). There were no individual longitudinal studies that skipped stages.

Because species with direct development do not have free‐swimming larvae and do not metamorphose, we analyzed those data in isolation. There were 260 studies that used species with direct development—most studies followed a cohort (46.9%) or focused on a single stage (51.9%) (Figures A1 and A2). Broadly, most studies began with measuring the F_0_ adult stage (Figure A3), and studies measuring sequential stages usually ended at the juvenile stage, meaning measurements of F_1_ adults were rare (Figure A4). However, there was one individual longitudinal study that measured all four stages (Figure A4c).

We also compared the relative frequency of the three development modes in our dataset to their frequency reported in the compilation from Marshall et al. (2012). Studies of planktotrophic species were overrepresented in our literature map—they were used in 62.3% of studies. Compared to Marshall et al. (2012), lecithotrophic species were underrepresented in articles by 23%, and direct developing species by 39.5%.

**FIGURE 5:**
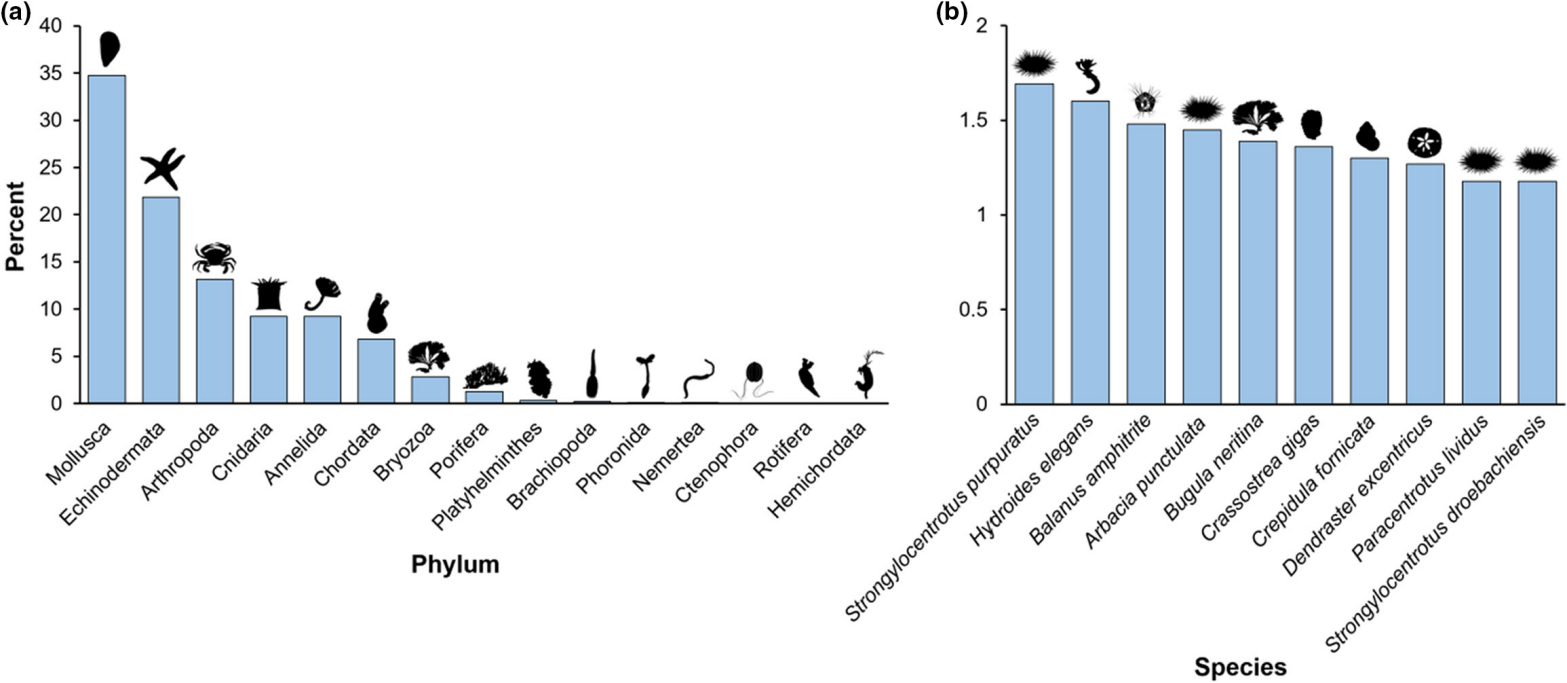
The taxa in the literature map of marine life histories (*n* = 3315 studies). (a) Percent of species in the 15 phyla—the three most common phyla accounted for ~70% of species. (b) The 10 most common species in the map—five are echinoderms: four sea urchins (*Arbacia punctulata*, *Paracentrotus lividus*, *Strongylocentrotus droebachiensis*, *S. purpuratus*) and one sand dollar (*Dendraster excentricus*).

The most common phyla studied were Mollusca (34.9%), Echinodermata (21.8%), and Arthropoda (13.1%), accounting for ~70% of the species in the map (Figure 5a). Of the 1225 resolved species in our dataset, the 10 most common species make up 13.8% of the dataset (n = 459 studies)—in other words, these 10 species were overrepresented by more than 17‐fold. Further, five of the 10 most common species were echinoderms: four sea urchins (*Arbacia punctulata*, *Paracentrotus lividus*, *Strongylocentrotus droebachiensis*, *Strongylocentrotus purpuratus*), and one sand dollar (*Dendraster excentricus*) (Figure 5b).

## 4 DISCUSSION

Our systematic map of marine invertebrate complex life histories identified emphases and knowledge gaps in the literature that affect our ability to understand correlations across stages. The most important issue our map identified was the lack of studies that both explore the entire life history and follow the same individuals over time. Instead, most studies focused on F_0_ adults, embryos or larvae, in isolation (67% of studies) or follow cohorts (31% of studies). Following individuals through the life cycle was rare (1.7% of studies), and no study measured the entire life history. The field has focused on describing single stages of life histories—it is tempting to infer correlations between two stages, even when they are studied as separate cohorts. But comparing two cohorts is inappropriate and highlights that most studies cannot make inferences about phenotypic correlations between stages. Studies that measured more than one stage almost exclusively followed cohorts, and could potentially be affected by Simpson's Paradox—at the very least, we are likely misestimating the strength of phenotypic correlations among life‐history stages and at worst, the true correlations could be the opposite of the patterns we observe among cohorts. If we are to better understand what phenotypic correlations exist and how they are relevant to fitness and population dynamics, then individual longitudinal studies should be a priority.

Importantly, we know other studies likely exist that meet some of our criteria (and some are even well‐known studies), but were not captured by our systematic map. This highlights an important limitation to systematic maps that must be acknowledged: no map will be perfectly comprehensive, so missing some important studies is unavoidable. Expanding search terms further generated an impractical number of papers to process, as the 17,000+ papers that we did screen required 100 s of person‐hours. Nevertheless, our map can be considered a representative and unbiased sample of the literature such that the relative abundance of different study types is unlikely to change, were broader terms used. Suffice it to say, regardless of how they are searched for, individual longitudinal studies remain rare in the literature.

Individual longitudinal studies are suggested as the best approach for addressing many life‐history questions including those on senescence, age‐related changes in the life history, population density regulation, selection, and phenotypic correlations between stages (Clutton‐Brock & Sheldon, 2010; Nussey et al., 2008). In comparison, using a cohort longitudinal approach can conceal individual‐level patterns in life‐history data. For example, an individual longitudinal study in the marine bryozoan *Bugula neritina* found that juvenile growth trajectories vary considerably across individuals—for the mean of the cohort, size increased through time, but at the individual level, some individuals grew, some shrank and others had no change, and these trajectories predicted reproductive output (Burgess & Bueno, 2021). We reemphasize a crucial point central to our argument regarding Simpson's paradox—patterns that are seen in a cohort may not reflect those in individuals. Individual longitudinal studies are the *only* methodology that reliably inform us about phenotypic correlations and their effect on selection and population dynamics (with the exception of quantitative genetic studies or when offspring traits of the entire brood are of interest, e.g., Aguirre et al., 2014; Cameron et al., 2017, 2021; Marshall, 2021). Thus, we suggest the field move toward the individual longitudinal approach (Cameron et al., 2019; Clutton‐Brock & Sheldon, 2010; Hoffmann & Sgró, 2011; Marshall, 2021, Schuster et al., 2021).

While we encourage using the individual longitudinal method, we acknowledge why studying individuals in the life history is rare—long‐term studies are costly, logistically difficult to maintain and, therefore, risky to undertake, particularly for long‐lived species (Clutton‐Brock & Sheldon, 2010). We found only one case in which all stages of the life cycle were measured—an individual longitudinal study that used a species with only four stages (i.e., direct development). Further, we found that cohort longitudinal studies measured the F_0_ adult, embryonic and larval stages most often (Figures 4a,b). However, we found the opposite trend in individual longitudinal studies, which measured metamorphs, juveniles, and F_1_ adults most often (Figure 4c,d). Why do studies following individuals mostly measure stages after metamorphosis? Of the studies that used an individual longitudinal approach, 71% used species with lecithotrophic larvae, which have relatively short development times. Culturing individuals with long larval stages is less straightforward—for example, it is much more difficult to study the whole life history of the planktotrophic sea star *Pisaster ochraceus*, which matures in 5 years (Menge, 1975), compared to the lecithotrophic marine bryozoan *Bugula neritina*, which takes ~7 weeks to mature (Keough, 1989). An interesting next step would be to explore whether there is also a dearth of individual longitudinal studies in terrestrial taxa (e.g., insects, frogs). Terrestrial groups have analogous life histories to marine invertebrates (e.g., indirect vs. direct development), so we expect those systems likely suffer from similar methodological limitations and biases, but this awaits testing.

Because of the challenge in executing individual longitudinal studies, phenotypic correlations between immature stages and F_1_ adults remain obscure—only 12 individual longitudinal studies in our map measured both juveniles and F_1_ adults, and none was done on species with long development times (i.e., planktotrophs). While planktotrophic species may be more difficult to study compared to lecithotrophs, there are excellent candidates for individual longitudinal studies that are in our dataset. For example, planktotrophic crustacean larvae, particularly decapods, are large, robust and have been cultured individually to the juvenile and/or adults stages in the laboratory (Oliphant et al., 2013; Oliphant & Thatje, 2013; Pansch et al. 2018; van Alstyne et al., 2014), and in the field (Lathlean & Minchinton, 2012). Our map suggests that rather than selecting species conducive to studying correlations between stages, we have prioritized studying a few model species that have small, less‐resilient larvae, and are not ideal for individual longitudinal studies (e.g., echinoderms; Figure 5). While model species, such as *Strongylocentrotus purpuratus* (echinoderm) or *Mytilus edulis* (mollusk), are important, going forward, we recommend that biologists select study species that can be tracked individually for the entire life cycle to gain a more holistic view of correlations between stages, and how they affect fitness.

Perhaps unsurprisingly, only 12% of studies in the dataset were conducted under field conditions, and of the field studies, less than 6% were individual longitudinal studies. The best examples of field‐based, individual longitudinal studies are in birds, primates, and other mammals—these studies are disproportionately represented in the literature, reflecting both how rare and difficult generating individual‐based studies in the field is, as well as how useful they are in answering ecological and evolutionary questions (Clutton‐Brock & Sheldon, 2010). Conducting studies in the field is important because tests in the laboratory can sometimes yield conflicting results—for example, guppies in a high predation environment had delayed senescence in the laboratory, but the pattern was the opposite when observed under natural conditions (Reznick & Ghalambor, 2005). Similarly, in a marine invertebrate, a laboratory experiment found there was the selection for mothers to produce small offspring, but in the field, there was the selection for small offspring in some cohorts and large in others (Monro et al., 2010). For marine invertebrates, studying certain life stages in the field may always be difficult, or may be restricted to certain species—for example, sessile species provide an opportunity to follow larvae to the juvenile and/or adult phase, because larvae can be settled on plates and then deployed in the field (Emlet & Sadro, 2006; Graham et al., 2013; Hettinger et al., 2013; Marshall et al., 2003; Phillips, 2002;). A more difficult task is following mobile species. There are a few examples of studies that followed free‐swimming embryos and larvae in the field (Young, 1986) or kept them in screened cages (Basch & Pearse, 1996; Nedelec et al., 2014), and a study that reared larvae in the laboratory and transplanted mobile juveniles to the field by using protective mesh (Li & Chiu, 2013). We encourage future studies to adapt and improve the methodologies we have discussed so that more species can be studied under natural conditions and we can understand the degree to which stages are linked.

Our systematic map shows that, for over 100 years, the field has done an excellent job in describing individual stages of marine invertebrate life histories, but this means that we have sacrificed understanding correlations between stages. Phenotypic correlations between stages have important implications for population dynamics (Burgess & Marshall, 2011; Taylor & Scott, 1997) and for how traits in different stages may evolve (Marshall & Morgan, 2011). While studies following cohorts are an important first step for answering questions about development, they risk Simpson's Paradox. The best approach for avoiding Simpson's Paradox is individual longitudinal studies, but these remain exceedingly uncommon, accounting for just 1.7% of studies in our map. While reviews in terrestrial systems have suggested that we move toward an individual longitudinal approach when studying life histories (Clutton‐Brock & Sheldon, 2010; Nussey et al., 2008), we are one of the first to systematically quantify the frequency of experimental approaches. We expected to find that individual longitudinal studies are rare, but we note that an important part of science is to confirm the gravity of the problem. We acknowledge and celebrate the tremendous progress in the field of complex life cycles, but we hope that the issues we have identified here encourages and incentivizes future studies to use an individual longitudinal approach to understand the ecological and evolutionary significance of phenotypic correlations across life‐history stages.

## AUTHOR CONTRIBUTIONS

**Emily L Richardson:** Data curation (lead); formal analysis (lead); investigation (lead); methodology (equal); visualization (lead); writing – original draft (lead); writing – review and editing (equal). **Dustin Marshall:** Conceptualization (lead); methodology (equal); project administration (lead); supervision (lead); validation (lead); visualization (equal); writing – original draft (supporting); writing – review and editing (equal).

## CONFLICT OF INTEREST STATEMENT

The authors declare no conflicts of interest.

## Data Availability

Data and code are archived and available on Dryad: https://doi.org/10.5061/dryad.3r2280gkj.

## APPENDIX A

**TABLE A1:** Inclusion and exclusion criteria for literature map of marine invertebrate life histories following the six‐stage Systematic Mapping Methodology from James et al. (2016).

Inclusion	Exclusion
Articles of marine invertebrates	Studies of vertebrates, freshwater, and terrestrial species
Empirical studies	Observational, qualitative, or theoretical studies
Measurements related to fitness (survival, size, growth, development time, and fecundity)	e.g., Behavior, metabolism, feeding rates, swimming rates

**TABLE A2:** Six stages identified in literature map of marine invertebrate life histories and the traits accepted for each.

Stage	Traits accepted
F_0_ adult	Body size; fecundity; egg size
Embryo	Fertilization success; hatching success; development time; survival; size; growth
Larva	Development time; size; growth; survival
Metamorph	Time to metamorphosis; size at metamorphosis; survival to metamorphosis
Juvenile	Development time; size; growth; survival; hatching success (direct developers)
F_1_ adult	Size; growth; fecundity

*Note*: Articles of marine invertebrates had to contain empirical data of fitness‐related traits for each stage. Development time for each stage is synonymous with “time to larval stage,” “time to maturity,”, etc.

**TABLE A3:** Search strings that were trialed during the scoping stage following the six‐stage Systematic Mapping Methodology from James et al. (2016) for the literature map of marine invertebrate life histories.

Search string	Hits
“Marine Invertebrate*” AND (Larva* OR Hatch* OR Fertili* OR Metamorph* OR Develop* OR Settle*)	~6000
Marine AND Invertebrate* AND (Larva* OR Hatch* OR Fertili* or Metamorph* OR Develop* OR Settle*)	~10,000
Marine AND Invertebrate* AND Larva* OR Hatch* OR Fertili* OR Metamorph* OR Develop* OR Settle*	~13,000,000
“Marine Invertebrate*” AND Larva* OR Hatch* OR Fertili* OR Metamorph* OR Develop* OR Settle*	~13,000,000
Marine AND Invertebrate* AND (Larva* OR Hatch* OR Fertili* OR Metamorph* OR Develop* OR Settle* OR “life history”)	~10,600
Marine AND Invert* AND (Larva* OR Hatch* OR Fertili* OR Metamorph* OR Develop* OR Settle* OR “life history” OR Plankto* OR Lecithotroph*)	~11,300

*Note*: In 2015, Web of Science, all databases were scoped for appropriate search terms—search terms were selected if ~10% of articles were accepted for the final literature map. Listed are search strings that were not used for this study.

**TABLE A4:** Summary of four searches conducted for the literature map of marine invertebrate life histories. For each search, the date, reviewer, and search terms used are listed. The number of hits is the total number of articles Web of Science returned for each search string. Articles were first screened at the title and abstract level, then relevant full‐text articles were assessed for eligibility. Articles meeting criteria (Tables A1 and A2) were retained for the map.

Search	Date	Reviewer(s)	Term	Hits	Title/abstract screened	Full‐text screened	Retained
1	6 Nov 2014	1	Topic: Larva* OR Hatch* OR Fertili* OR Metamorph* OR Develop* OR Settle* AND Publication name: Biological Bulletin	1873	1873	1674	225
2	10 Dec 2014	1	Topic: Larva* OR Hatch* OR Fertili* OR Metamorph* OR Develop* OR Settle* AND Publication name: Marine Biology	2629	1000	680	120
3	20 July 2015	2,3	Topic: Marine AND Invert* AND (Larva* or Hatch* or Fertili* or Metamorph* or Develop* or Settle* or “life history” or Plankto* or Lecitho* or Embryo* or Gamet* or Egg* or Sperm* or Juvenil*)	12,372	12,372	3391	1035
4	29 Jun 2021	4	Topic: Marine AND Invert* AND (Larva* or Hatch* or Fertili* or Metamorph* or Develop* or Settle* or “life history” or Plankto* or Lecitho* or Embryo* or Gamet* or Egg* or Sperm* or Juvenil*) AND Year Published: 2016–2021	2333	2333	807	336
